# A comparison of the real world effectiveness of catheter ablation and drug therapy in atrial fibrillation patients in a Chinese setting

**DOI:** 10.1186/s12872-017-0634-y

**Published:** 2017-07-27

**Authors:** Xin Du, Lizhu Guo, Xiaonan He, Yu Jia, Jiahui Wu, Deyong Long, Ronghui Yu, Caihua Sang, Xiaohui Liu, Hongjun Yin, Jianwei Xuan, Jianzeng Dong, Changsheng Ma

**Affiliations:** 1 Beijing Anzhen Hospital, Capital Medical University, No 2 Anzhen Road, Chaoyang District, Beijing, 100029 China; 2 Strategic Medical Affairs, Johnson & Johnson Medical (China) Ltd., Shanghai, China; 3 Shanghai Centennial Scientific Ltd., Inc., Shanghai, China; 4 Health Economic Research Institute, Sun-Yat-sen University, Zhongshan, Guangdong China

**Keywords:** Catheter ablation, Atrial fibrillation, Anti-arrhythmic drugs, Quality of life, Chinese

## Abstract

**Background:**

Studies have demonstrated that catheter ablation of atrial fibrillation is associated with better rhythm control than drug therapy. The present study aimed to assess the clinical outcomes and health-related quality of life of ablation therapy in a real world setting.

**Methods:**

A prospective, non-randomized, single center study in a real-world clinical setting in China was conducted. Patients were followed up at 3, 6, and 9 months after baseline encounter. Propensity score matched patients receiving ablation or anti-arrhythmic drug therapy were compared. Incidence rate of atrial fibrillation recurrence and quality of life outcomes were measured and analyzed using log-rank test, multivariate logistic regression and mixed-effects linear regression respectively.

**Results:**

In this study, 151 atrial fibrillation patients treated by ablation therapy and 318 patients treated by anti-arrhythmic drugs were enrolled. During follow up, 82.0% in the ablation arm and 22.4% in the drug arm had no documented atrial fibrillation recurrence [HR for atrial fibrillation recurrence 0.07 (95%CI: 0.02–0.21, *p* < 0.0001)] among paroxysmal atrial fibrillation patients. The corresponding no recurrent rate were 66.7% and 18.5% [0.21 (0.05–0.95, *p* = 0.04)] respectively among persistent atrial fibrillation patients. Improvement in Short Form-36 physical component scores, Short Form-36 mental component scores and total Atrial Fibrillation Effect on Quality-of-life scores were 16.33 (14.05–18.61, *p* < 0.001), 8.10 (6.11–10.09, *p* < 0.001) and 18.28 (16.11–20.45, *p* < 0.001) respectively among paroxysmal AF patients and 6.32 (3.15–9.49, *p* < 0.001), 3.99 (1.82–6.16, *p* < 0.001) and 13.97 (10.89–17.05, *p* < 0.001) respectively among persistent AF patients. Improvements in total Atrial Fibrillation Effect on Quality-of-life score were also significant in ablation arm while no significant improvement of total Atrial Fibrillation Effect on Quality-of-life score in the drug arm.

**Conclusion:**

Compared with drug therapy, catheter ablation is associated with significant lower AF recurrence and improved overall quality of life.

**Trial registration:**

The present study has been registered on clinicaltrials.gov. The ClinicalTrials.gov ID is NCT01878981. The registration date is May 29, 2013.

## Background

Atrial fibrillation (AF) is independently associated with increased risk of all-cause mortality in women (two times) and in men [1, 2] and increased morbidity (heart failure and stroke) and cardiovascular mortality. It also causes significant adverse impact on patients’ quality of life [3]. Rhythm control, rate control and anticoagulation therapy are the three most important pillars of treatment. In recent years, catheter ablation has been widely used for AF patients as a strategy of rhythm control. This is known to be associated with significantly better effectiveness compared with antiarrhythmic drugs in lowering rate of recurrent atrial tachyarrhythmias while the results are conflict with whether ablation therapy can improvement patient’s quality of life [4–6]. The evidence for cardiovascular outcome improvement is still lacking. There are substantial geographical variations in the management of AF and experience are growing in refining clinical indications for ablation therapy [7].

Catheter ablation for AF has been widely applied in China. However, available studies on the clinical outcome and quality of life (QoL) of catheter ablation compared with drug therapy in real world setting are sparse in this population. This study aimed to report the real world comparative effectiveness of these two treatment strategies.

## Methods

### Study design

The design is a prospective, non-randomized, single center study in a real-world clinical setting in Anzhen Hospital, a referral hospital in Beijing, China. The study was approved by the Ethics Committee of Anzhen Hospital and informed consent was obtained from the patients included. Medical practices in the hospital are representative of hospitals in the same class in China. Participating physicians were given the discretion to decide whether a patient should receive ablation procedures or anti-arrhythmia therapy provided they followed local and international guidelines.

### Patients

Patients were enrolled over approximately 2 years beginning in the second quarter of 2011. Inclusion criteria in the study were paroxysmal or persistent AF patients eligible for catheter ablation, age 18 to 80 years, willing to comply with all face to face follow-up visiting and requirements, and sign the informed consent form.

Candidates with any of the following conditions were excluded from the study: terminal illness with a life expectancy <1 year, patients with symptomatic heart failure (New York Heart Association Class III or IV), previous recipient of catheter ablation therapy for AF, bradycardia (heart rate < 60 beat per minutes) and previous recipient of pacemaker therapy, uncontrolled hypertension (blood pressure > 140/90 mmHg despite three different full dosage anti-hypertensive therapy), recent cardiac events including myocardial infarction, percutaneous coronary intervention or valve or bypass surgery in the preceding 3 months, serious hepatic disease (hepatic biomarkers higher than 5 times upper normal limits) and renal dysfunction (estimated GFR ≤ 60 ml/min), pregnant or preparing to be pregnant within one year. In addition, patients who could not be contacted via phone (despite 3 documented attempts to contact the subject). Patients that were enrolled but never underwent ablation or took any AAD were also excluded.

Patients who met the inclusion criteria and signed the patient informed consent form were given a 30-day window (±7 days) to make a final decision regarding participation in the study.

Data of demographic characteristics, social economic variables and clinically related comorbidities were collected based on the patient reported information.

### Ablation procedures or anti-arrhythmia therapy

The ablation procedures include pulmonary vein (PV) isolation for paroxysmal AF, and three additional linear ablations at the left atrial roof, mitral isthmus between the mitral annulus and left inferior PV and cavotricuspid isthmus for persistent AF. Antiarrhythmic agents were not allowed to be used after ablation procedure. Patients were asked to check their ECG if they felt palpitation or any symptom of AF episode. Otherwise, 24 h ECG monitoring will be employed every month. For patients without evidence for AF recurrence, oral anticoagulants can be withdrawn 3 months after the procedure, regardless their baseline risk of stroke according to CHA_2_DS_2_-VASc score.

Medical therapy was at the discretion of responsible physician. Anti-arrhythmia medicines include sotalol 80 mg every 8 h, with a maximum dosage of 320 mg/day, propafenone 150 mg every 8 h, and amiodarone 600 mg/day for the first week, 400 mg/day for the next week, and 200 mg a day after that. Usage of cardioversion was also at the discretion of the physician. Any cardioversion received during study period was recorded. Anticoagulation therapy will be provided according to guideline recommendation.

### Follow-up

Face to face follow-up occurred at 3, 6, and 9 months, after the 3 months blanking period in the ablation group and after baseline enrollment in the drug arm.

### Outcomes

The primary clinical endpoint was the incidence rate of AF recurrence within the 9-month follow-up period starting immediately after the blanking period for the ablation arm or after baseline enrollment for the drug arm. The “AF recurrence episode” was defined as an episode of atrial fibrillation/atrial flutter/atrial tachycardia ≥30 s in duration as documented by ECG, Holter monitor, or telemetry recording. Three failure modes of the primary endpoint were considered: first, acute ablation procedural failure; second, a new AAD for AF during the 9-month follow-up period; and third, a repeat ablation during the 9-month follow-up period post-blanking.

Patients’ QoL was measured using both a generic instrument, Short Form-36 (SF-36), and a disease-specific instrument, Atrial Fibrillation Effect on Quality-of-life (AFEQT). SF-36 is a validated instrument that measures an individual’s physical and mental health [8]. The Physical Component Summary (PCS) and Mental Component Summary (MCS) were calculated using norm-based scoring according to the guideline from Quality Metric Incorporated. The absolute changes from baseline of PCS and MCS at each follow-up visit (at 3, 6, 9 and 12- month) were summarized and plotted for each treatment group. A minimum 3-point improvement in SF-36 subscales is deemed to be clinically important in patients with chronic diseases. The proportion of subjects with greater than or equal to a 3-point improvement in MCS and PCS was also reported at baseline and follow-up visits. The analysis population was propensity score matched.

The AFEQT is another validated AF-specific QoL instrument utilized in the study, which is comprised of 20 questions [9]. Patients answered the questionnaire based on their memory or feeling of the previous four weeks. The total score and three subscale scores of symptoms, daily activities and treatment concern were summarized by treatment group at each follow-up visit (at 3, 6, 9 and 12- month). The absolute changes from baseline of the overall and subscale scores at each follow-up visit were summarized and plotted for each treatment group. The overall or subscale scores range from 0 to 100. A score of 0 corresponds to complete disability (or responding “extremely” limited, difficult or bothersome to all questions answered), while a score of 100 corresponds to no disability (or responding “not at all” limited, difficult or bothersome to all questions answered).

### Statistical analysis

A priori power analysis was conducted based upon survival log-rank test, accounting for the varying follow-up times that the ablation patients may have. The assumptions include a length of enrollment of 1.5 years, maximum length of follow-up of 2.5 years, a 10% drop-out rate and 30% success rate for drug therapy among both paroxysmal and persistent patients. It was further assumed that ablation success rate was 60% for paroxysmal patients and 45% for persistent patients, which were calculated based upon success rate, drop-out rate, and maximum follow-up time [10]. It was assumed that there would be a 10% crossover rate for each AF type. Based upon the calculation, at least 53 pairs were needed to compare ablation versus AAD treatment for the paroxysmal patients and at least 200 pairs were needed for persistent patients to ensure 80% power (alpha = 0.05, two-tailed).

The primary clinical endpoint is the frequency of recurrence of atrial fibrillation/atrial flutter/atrial tachycardia within the 9 months follow up period starting immediately after the blanking period for ablation arm or baseline enrollment for drug arm. To examine the corresponding relative risk, we used time to event analysis (e.g. crude Kaplan-Meier analysis, Cox model stratified by matched pairs with partial likelihood estimation [11, 12]. The analysis population was the propensity score (PS)-matched population.

Optimal pair matching based on logit of the PS created with a matching ratio of 1:1, stratified by AF type was used in this study. Optimal matching identifies matched sets in such a way that the process aims to optimize the total distance rather than distance of an individual pair. A big advantage of optimal matching over more traditional greedy matching is that the former approach could generate more desirable matched pairs. Candidate covariates considered for the PS analysis included patients’ demographic characteristics and social economic variables as well as certain clinically related comorbidities, most of which are presented in Table 1. Catheter ablation patients were matched with AAD patients by a 1:1 ratio to ensure the comparability of patients enrolled in each arm at baseline. PS matching was performed for each AF type separately and the analysis was stratified by the two AF types. Based upon the PS-matched patients, log-rank test and multivariate logistic regression were conducted to compare the effects on AF recurrence. Mixed-effects linear regression with interaction between time and treatment grouping variables was utilized to examine potential difference in QoL trends over time between the treatment groups. The mix-effects model can account for the dependency inherent in the data due to repeated measurements and allow respondents with missing observations at different time points [13]. A *p* value ≤0.05 is deemed statistically significant.

**Table 1:** Baseline characteristics before and after matching by treatment

	Paroxysmal AF patients						Persistent AF patients					
	Before matching (n/N)			After matching (n/N)			Before matching (n/N)			After matching (n/N)		
Characteristics	Ablation	Drug	Sig.	Ablation	Drug	Sig.	Ablation	Drug	Sig.	Ablation	Drug	Sig.
Age (≥75 yrs)	5.33% (4/75)	17.90% (29/162)	*	5.33% (4/75)	5.33% (4/75)		26.32% (20/76)	57.69% (90/156)	*	35.09% (20/57)	35.09% (20/57)	
Sex (Male)	57.33% (43/75)	59.26% (96/162)		57.33% (43/75)	62.67% (47/75)		73.68% (56/76)	57.69% (90/156)	*	68.42% (39/57)	57.89% (33/57)	
BMI (≥25)	49.33% (37/75)	51.23% (83/162)		49.33% (37/75)	56.00% (42/75)		76.32% (58/76)	53.21% (83/156)	*	68.42% (39/57)	68.42% (39/57)	
Coronary Atherosclerotic Heart Disease (yes)	10.67% (8/75)	16.67% (27/162)		10.67% (8/75)	16.00% (12/75)		5.26% (4/76)	19.23% (30/156)	*	3.51% (2/57)	3.51% (2/57)	
NYHABA (>Level I)	6.67% (5/75)	12.35% (20/162)		6.67% (5/75)	6.67% (5/75)		2.63% (2/76)	9.62% (15/156)		3.51% (2/57)	3.51% (2/57)	
Other Arrhythmia (yes)	10.67% (8/75)	4.94% (8/162)		10.67% (8/75)	5.33% (4/75)		2.63% (2/76)	2.56% (4/156)		3.51% (2/57)	1.75% (1/57)	
Hypertension (yes)	60.00% (45/75)	59.88% (97/162)		60.00% (45/75)	62.67% (47/75)		56.58% (43/76)	50.00% (78/156)		56.14% (32/57)	45.61% (26/57)	
Thrombotic diseases (yes)	13.33% (10/75)	15.43% (25/162)		13.33% (10/75)	17.33% (13/75)		9.21% (7/76)	15.38% (24/156)		12.28% (7/57)	17.54% (10/57)	
Diabetes (yes)	13.33% (10/75)	17.28% (28/162)		13.33% (10/75)	20.00% (15/75)		10.53% (8/76)	14.74% (23/156)		10.53% (6/57)	17.54% (10/57)	
Bleeding (yes)	2.67% (2/75)	2.47% (4/162)		2.67% (2/75)	1.33% (1/75)		1.32% (1/76)	3.21% (5/156)		1.75% (1/57)	1.75% (1/57)	
Chronic Bronchitis and Chronic Obstructive Pulmonary Disease (yes)	0.00% (0/75)	8.02% (13/162)	*	0.00% (0/75)	10.67% (8/75)	*	2.63% (2/76)	7.69% (12/156)		3.51% (2/57)	7.02% (4/57)	
Hyperthyroidism (yes)	1.33% (1/75)	0.62% (1/162)		1.33% (1/75)	1.33% (1/75)		98.68% (75/76)	95.51% (149/156)		98.25% (56/57)	94.74% (54/57)	
Education (High School or less)	48.00% (36/75)	54.32% (88/162)		48.00% (36/75)	61.33% (46/75)		43.42% (33/76)	70.51% (110/156)	*	57.89% (33/57)	57.89% (33/57)	
Insurance Status												
1	86.67% (65/75)	88.27% (143/162)		86.67% (65/75)	85.33% (64/75)		92.11% (70/76)	85.26% (133/156)		92.98% (53/57)	84.21% (48/57)	
2	13.33% (10/75)	11.73% (19/162)		13.33% (10/75)	14.67% (11/75)		7.89% (6/76)	14.74% (23/156)		7.02% (4/57)	15.79% (9/57)	
Monthly Family Total Income												
< 2000	0.00% (0/75)	6.17% (10/162)		0.00% (0/75)	5.33% (4/75)		3.95% (3/76)	3.85% (6/156)		3.51% (2/57)	5.26% (3/57)	
2000–5000	37.33% (28/75)	27.16% (44/162)		37.33% (28/75)	34.67% (26/75)		26.32% (20/76)	33.97% (53/156)		31.58% (18/57)	28.07% (16/57)	
5000–10,000	44.00% (33/75)	40.74% (66/162)		44.00% (33/75)	42.67% (32/75)		40.79% (31/76)	33.33% (52/156)		40.35% (23/57)	28.07% (16/57)	
> 10,000	18.67% (14/75)	25.93% (42/162)		18.67% (14/75)	17.33% (13/75)		28.95% (22/76)	28.85% (45/156)		24.56% (14/57)	38.60% (22/57)	
Family Monthly Income (RMB)												
< 1000	1.33% (1/75)	6.79% (11/162)		1.33% (1/75)	6.67% (5/75)		3.95% (3/76)	3.21% (5/156)		3.51% (2/57)	5.26% (3/57)	
1000–2500	36.00% (27/75)	29.01% (47/162)		36.00% (27/75)	34.67% (26/75)		27.63% (21/76)	36.54% (57/156)		35.09% (20/57)	29.82% (17/57)	
2500–5000	53.33% (40/75)	52.47% (85/162)		53.33% (40/75)	54.67% (41/75)		51.32% (39/76)	44.23% (69/156)		45.61% (26/57)	36.84% (21/57)	
> 5000	9.33% (7/75)	11.73% (19/162)		9.33% (7/75)	4.00% (3/75)		17.11% (13/76)	16.03% (25/156)		15.79% (9/57)	28.07% (16/57)	

*p ≤ 0.05

## Results

### Characteristics of patients

**Fig. 1:**
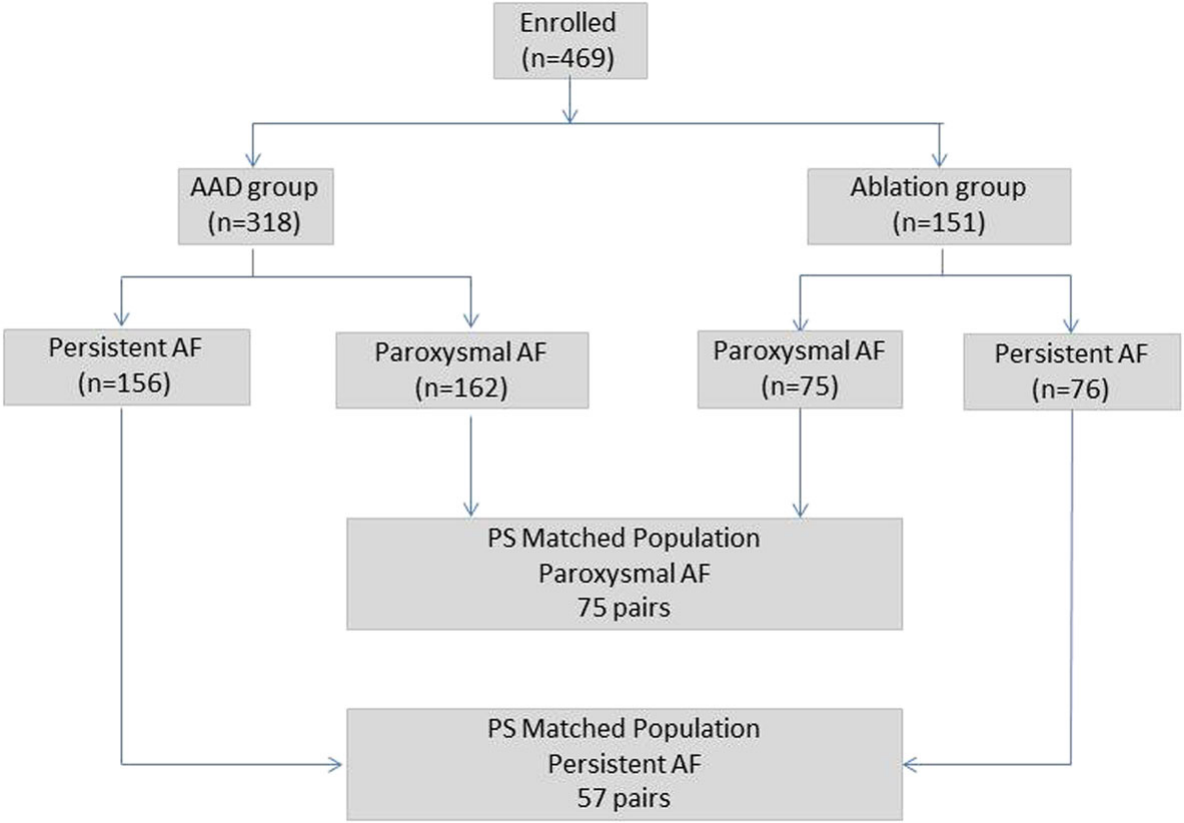
Flowchart of Patient Selection. Abbreviations: AAD: antiarrhythmia drugs; AF: atrial fibrillation; PA: propensity score

In total, 469 patients were enrolled into the study: 151 in the ablation group (75 subjects had paroxysmal AF and 76 subjects had persistent AF) and 318 in the drug group (162 subjects had paroxysmal AF and 156 subjects had persistent AF). In the AAD arm, 6 patients did not take any AAD drug and were therefore excluded from the study, 26 subjects withdrew within one month of the study and 3 additional patients withdrew before the 9-month visit. In the ablation arm, 9 patients had the catheter inserted but did not receive any RF delivery and 3 patients withdrew early. Patients enrolled in the ablation arm and AAD arm had similar echocardiographic data before matching (left ventricular ejection fraction: 64.38 ± 7.27 vs 64.00 ± 4.58). Propensity score matching resulted in 75 pairs of paroxysmal AF subjects and 57 pairs of persistent AF subjects (Fig. 1). Table 1 compares the ablation and drug arms before and after the PS matching. After matching, all the characteristics in the two treatment arms were similar except proportion of patients with chronic bronchitis or obstructive pulmonary diseases remained significantly different among paroxysmal AF patients.

### Clinical outcomes

**Fig. 2:**
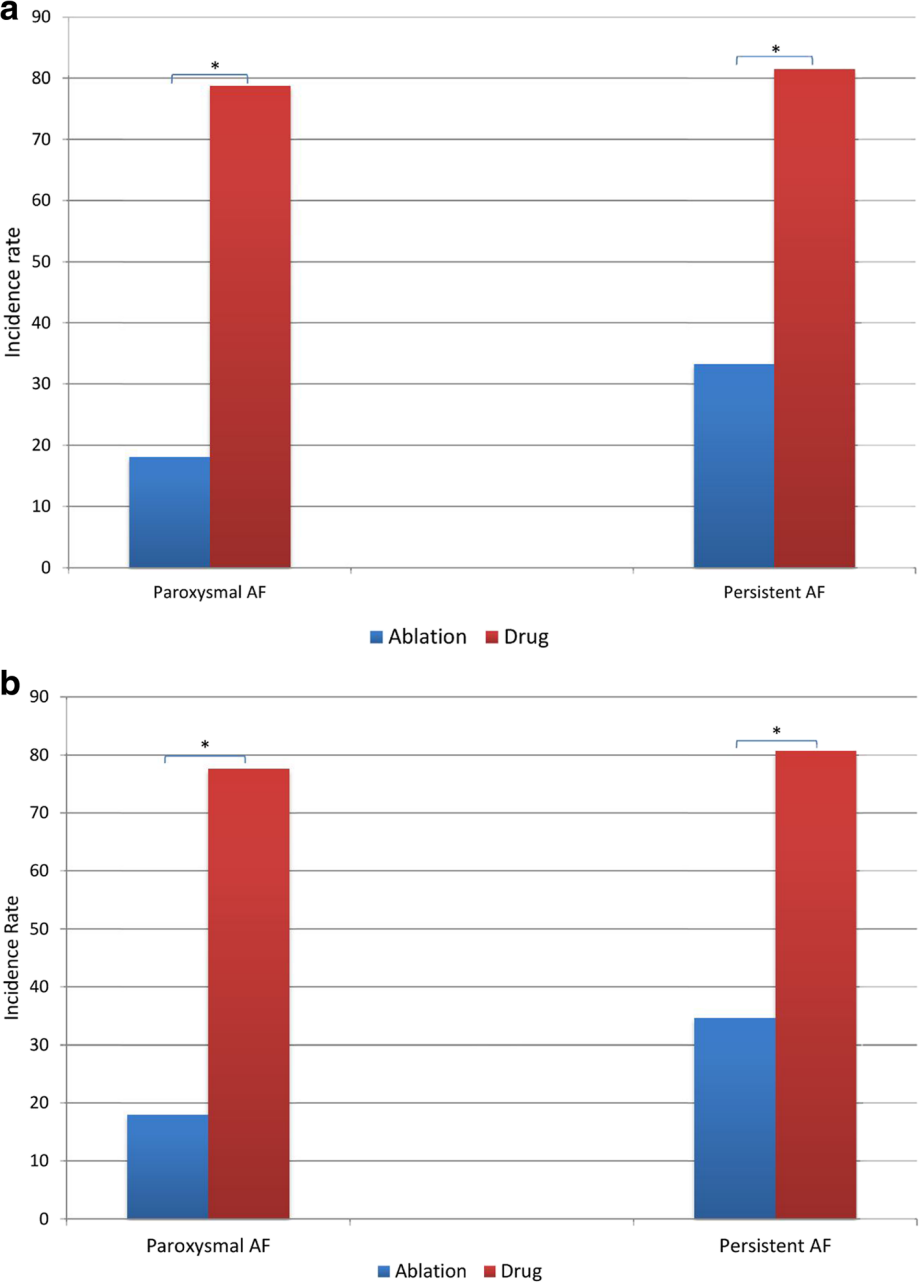
Rates of AF recurrence in the ablation and drug arms * *p* ≤ 0.05. **a** Before matching. **b** After matching

**Fig. 3:**
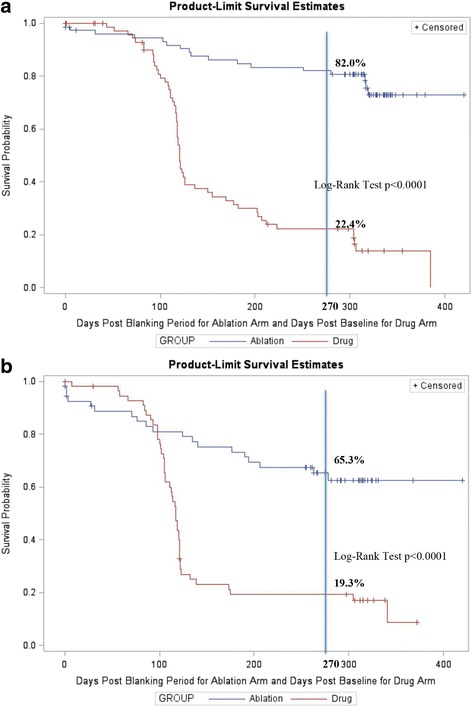
Freedom from Documented AF/AFL/AT Recurrences with Failure Modes for Matched Patients. **a** Paroxysmal AF patients. **b** Persistent AF Patients

Among patients with paroxysmal AF, incidence rate of recurrence was 18.0% (95%CI: 10.0–28.9%) in the ablation arm and 77.6% (95%CI: 67.0–87.9%) in the drug arm (Fig. 2a). For matched paroxysmal AF subjects, the ablation arm had significantly higher success rate than the drug arm (78.5% vs 18.0%, log-rank test *p*-value < 0.001) (Figs. 2b and 3a). In post-matching conditional logistic regression analyses stratified by paroxysmal or persistent AF and controlling for clinically important covariates, the hazard ratio of AF recurrence in the ablation arm compared to the drug arm was 0.070 (95%CI: 0.023–0.214)(*p* < 0.0001).

Among patients with persistent AF, incidence rate of recurrence was 33.3% (95%CI: 21.1–47.5%) in the ablation arm and 81.5% (95%CI: 68.6–90.7%) in the drug arm (Fig. 2a). For matched persistent AF subjects, the ablation arm had significantly higher success rate of than the drug arm (80.9% vs 35.1%, log-rank test *p*-value < 0.0001) (Figs. 2b and 3b). In post-matching conditional logistic regression analyses stratified by paroxysmal or persistent AF, and controlling for clinically important covariates, the hazard ratio of AF recurrence in the ablation arm compared to the drug arm was 0.209 (95%CI:0.046–0.952) (*p* = 0.04).

### QoL: SF-36 scores among AF patients

**Fig. 4:**
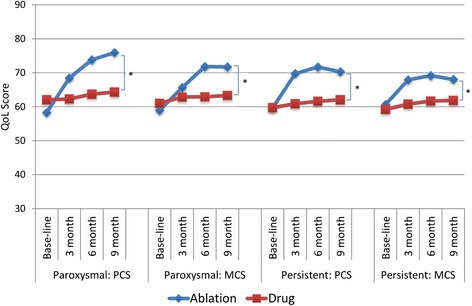
Change in SF-36 Physical and Mental Component Scores among the Ablation and Drug Patients. PCS stands for Physical Component Score. MCS stands for Mental Component Score. * *p* ≤ 0.05

Among matched patients with paroxysmal AF, the ablation subjects showed significant improvement in their QoL physical component scores (PCSs) measured by SF-36 at all 3-, 6- and 9-month visits post blanking (*p* < 0.001) (Fig. 4). The score at baseline was at 58.31 while improved to 75.92 at 9-month visit, a 30.2% increase. On the contrary, the PCSs of the subjects in the drug arm only increased from 62.05 to 64.31 at the 9-month visit, which was not statistically significant. Significant improvement to 71.72 (21.7%) in QoL mental component scores (MCS) measured by SF-36 was also observed in the ablation arm (*p* < 0.01) during the study period. In contrast, the MCS of the subjects in the drug arm increased only from 60.98 to 63.30 at 9-month visit (*p* = 0.03).

Among matched patients with persistent AF, the ablation arm showed significant improvement in their QoL PCSs at all 3-, 6- and 9-month visits post blanking (*p* < 0.001). The scores changed by 17.8%, from 59.67 at baseline to 70.28 at 9-month visit. On the contrary, the PCSs of the subjects in the drug arm increased only slightly from 59.72 to 62.06 during same study, which was not significant. The QoL MCSs score was improved by 12.1% from 60.66 at baseline to 68.01 at 9-month visit in the ablation arm (*p* < 0.05), while the MCSs of the subjects in the drug arm remained unchanged (from 59.2 to 61.84, *p* = 0.14).

For both the paroxysmal and persistent AF patients, the mixed-effects linear regression showed that the changes of PCS and MCS over time in the ablation arm were significantly different from those in the drug arm (p all <0.05, Fig. 4).

### QoL: AFEQT scores among AF patients

Among patients with paroxysmal AF, total AFEQT scores in the ablation arm improved from 54.4 at baseline to 79.19 at 9-month visit showing a 45.6% increase (*p* < 0.001). In the AAD arm, the total AFEQT scores changed slightly from 63 at baseline to 64.89 at 9 months visit (*p* = 0.17). The improvement in the AFEQT subscale scores of symptoms in ablation arm was much larger than in the drug arm (48.2% vs 4.9% change from baseline) (*p* < 0.001). Changes of AFEQT subscale scores of daily activities and treatment concerns over time in the ablation arm were also significantly different from those in the drug arm (*p* < 0.001).

Among patients with persistent AF, total AFEQT scores of the ablation arm increased by34.5%, from 55.4 at baseline to 74.5 at 9-month visit (*p* < 0.001) while remained stable in the drug arm (from 61.0 at baseline to 64.4 at 9-month visit, *p* = 0.07). The AFEQT subscale scores of symptoms, daily activity and treatment concerns all increased significantly in the ablation arm; In contrast, neither of the AFEQT subscale symptom score nor daily activities score showed significant change in the drug arm. Only the subscale scores of treatment concerns improved by 9.1% in the drug arm during the same time period (*p* = 0.01).

**Fig. 5:**
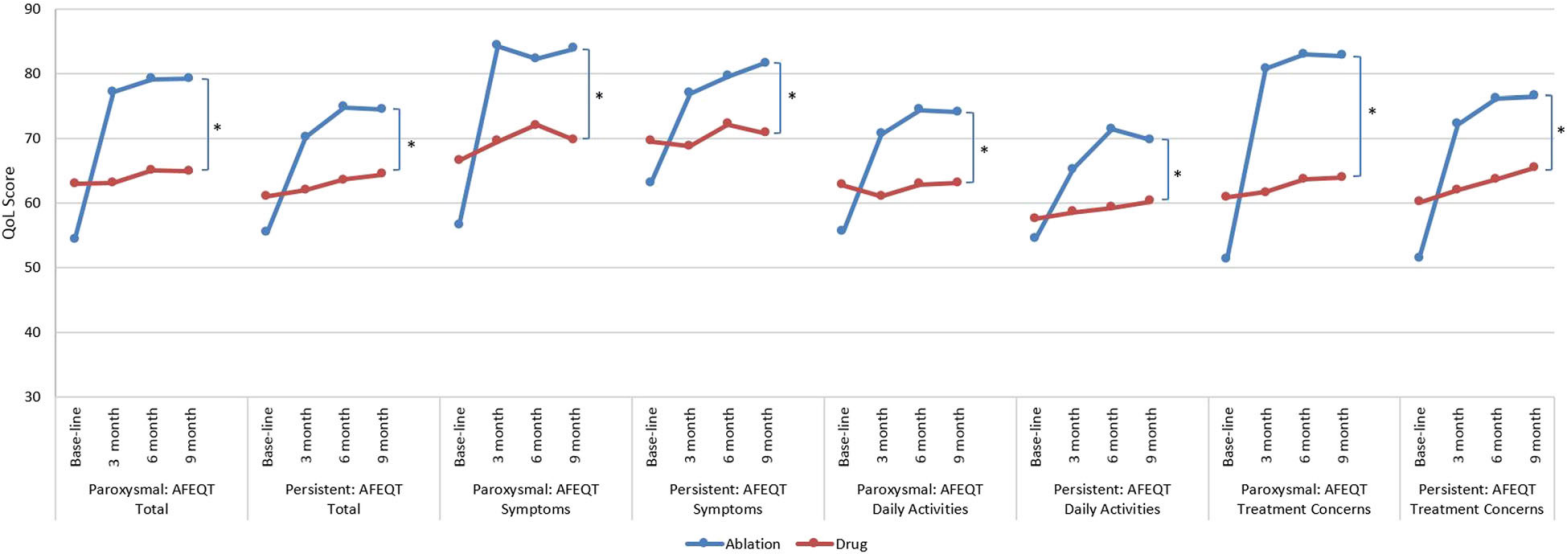
Change in AFEQT Total and Subscale Scores among the Ablation and Drug Patients * *p* ≤ 0.05

Among both paroxysmal and persistent AF patients, based on the mixed-effects linear regression the changes of total AFEQT scores and subscale scores over time in the ablation arm were significantly different from those in the drug arm (*p* < 0.001, Fig. 5).

## Discussion

In this study, we found that patients treated with catheter ablation had lower risk of AF recurrence than those treated with AADs for both paroxysmal and persistent AF patients. The difference was statistically significant and clinical meaningful.

Among paroxysmal or persistent patients, the ablation arm had significantly better quality of life improvement than the AAD arm as measured with SF-36 and AFEQT. Specifically, the ablation arm had consistent and significant improvement in physical and mental scores measured with SF-36, while the AAD arm showed only a small improvement during the follow-up time periods. Similar patterns were observed in the change in AFEQT total and subscale scores. It should be noted that improvement in different dimensions varied when measured by both instruments, among both paroxysmal and persistent patients.

It is consistently reported that catheter ablation was more effective than AADs in reducing the risk of AF recurrence [3–5, 14]. However, the majority of studies only included patients with paroxysmal AF and study examining the impact of treatment on patients’ quality of life is scarce. The present study supports the superiority of catheter ablation to AAD using real-world data from China. It showed that catheter ablation was more effective than drug therapy in reducing recurrence of AF and improving patients’ quality of life.

The evidence generated by this real-world study is expected to help inform medical decision makers about the effectiveness of two important treatments for AF patients. Given the demonstrated superior efficacy in maintaining sinus rhythm and important improvement in QoL with ablation, our results support using of catheter ablation in patients similar to those enrolled in this study.

### Limitations

Several study limitations should be noted. First, the effectiveness of the ablation treatment at the 9-month visit seemed to be higher than normally observed. Possible reasons include (i) this study was carried out in a single center with more experience in providing ablation therapy; (ii) under report of recurrences due to lack of rigorous monitoring and under-reporting from the patients. Second, not all of the subjects in the drug therapy arm persistent to AADs therapy during the 12-month follow-up period. However, it is less likely to achieve a higher rate of treatment success in patients who have AF recurrent on AADs treatment. Third, the follow-up time period of 9 months was relatively short. The long-term effectiveness and persistence of QoL improvement cannot be captured in this study.

## Conclusions

The present study found that catheter ablation resulted in significantly better effectiveness for both paroxysmal and persistent AF patients during the 9-month follow-up period. The ablation treatment has also shown considerably improved quality of life outcomes compared with drug therapy. Slightly more adverse events were observed in patients with ablation therapy and the complete benefit and cost evaluation are important area for further study.

## Abbreviations

AADs: Antiarrhythmic drugs
AF: Atrial fibrillation
AFEQT: Atrial Fibrillation Effect on Quality-of-life
MCS: (SF-36) Mental Component Summary
PCS: (SF-36) Physical Component Summary
PV: Pulmonary vein
QoL: Quality of life
SF-36: Short Form-36
